# Supplementary material for: Carbon emissions from the 2023 Canadian wildfires
Source: Nature. 2024 Aug 28;633(8031):835–9. doi: 10.1038/s41586-024-07878-z (PMC11424480; doi:10.1038/s41586-024-07878-z)
Supplement: Supplementary file 1 — Supplementary sections 1 and 2, Figs. 1–14, Tables 1–3 and references. [file 41586_2024_7878_MOESM1_ESM.pdf]

nature portfolio

https://doi.org/10.1038/s41586-024-07878-z

Supplementary information

Carbon emissions from the 2023 Canadian wildfires

In the format provided by the authors and unedited

Supplementary information for "Carbon emissions from the 2023 Canadian Wildfires"

Brendan Byrne[1*], Junjie Liu[1,2], Kevin W. Bowman[1,3], Madeleine Pascolini-Campbell[1], Abhishek Chatterjee[1], Sudhanshu Pandey[1], Kazuyuki Miyazaki[1], Guido R. van der Werf[4], Debra Wunch[5], Paul O. Wennberg[2,6], Coleen M. Roehl[2], Saptarshi Sinha[7]

[1*]Jet Propulsion Laboratory, California Institute of Technology, Pasadena, CA, USA.
[2]Division of Geological and Planetary Sciences, California Institute of Technology, Pasadena, CA, USA.
[3]Joint Institute for Regional Earth System Science and Engineering, University of California, Los Angeles, CA, USA.
[4]Meteorology & Air Quality Group, Wageningen University and Research, Wageningen, The Netherlands.
[5]Department of Physics, University of Toronto, Toronto, ON, Canada.
[6]Division of Engineering and Applied Science, California Institute of Technology, Pasadena, CA, USA.
[7]Department of Energy, Environmental, and Chemical Engineering, Washington University, St. Louis, MO, USA.

*Corresponding author(s). E-mail(s): brendan.k.byrne@jpl.nasa.gov; Contributing authors: junjie.liu@jpl.nasa.gov; kevin.w.bowman@jpl.nasa.gov; madeleine.a.pascolini-campbell@jpl.nasa.gov; abhishek.chatterjee@jpl.nasa.gov; sudhanshu.pandey@jpl.nasa.gov; kazuyuki.miyazaki@jpl.nasa.gov; guido.vanderwerf@wur.nl; dwunch@atmosp.physics.utoronto.ca; wennberg@caltech.edu; coleen@caltech.edu; sinha.s@wustl.edu;

.

S1 Detailed description of inversion results

We performed a large number of individual inversion that differ in prior fire inventory and inversion configuration. Here, we provide more details on the posterior flux estimates from these inversions. Figure S3 shows the prior and posterior fluxes for each inversions configuration and inventory. We find that the different inversion configurations only have a modest impact on the posterior fluxes, with the $CO+CO_2$ May-Sep fire emissions ranging over 589–693 TgC. The Bayesian posterior uncertainties are also shown for the inversions with 7 day optimization and include observational representativeness errors, which give a 1 σ uncertainty of 23–56 TgC. The spread among inversion configurations and Bayesian posterior errors are added in quadrature to give total errors for the inversion ensemble.

The abundance of the hydroxyl radical (OH) controls the loss of OH, and therefore strongly impacts emission estimates. In this study, we use OH output by the MOMO-Chem chemical data assimilation system, which has undergone extensive evaluation [1, 2]. Still, the atmospheric abundance of OH is uncertain, so here we provide an assessment of the sensitivity of emission estimates to prescribed OH. This is tested by re-running the full set of experiments using different OH fields [3] (referred to as "GEOS-Ghem"). Figure S1 shows that the lower air-mass-weighted OH concentrations produced by the GEOS-Chem simulation (10.3×10^5 molec cm^{-3}) results in a 37% reduction in estimated $CO+CO_2$ emissions relative to the MOMO-Chem OH abundance (11.4×10^5 molec cm^{-3}). For comparison, the distribution of modeled OH abundances for the Atmospheric Chemistry and Climate Modeling Intercomparison Project (ACCMIP) [4]. A further complication is that both OH abundances and atmospheric CO production are impacted by fires. This sensitivity analysis emphasizes the importance of OH abundances of fire emission estimates, and motivates further work to better quantify OH fields.

S2 Performance of atmospheric CO inversions

The performance of the atmospheric CO inversions is evaluated by investigating the mismatch of posterior CO fields against the assimilated TROPOMI X_{CO} retrievals and independent TCCON X_{CO} retrievals. The mismatch against assimilated TROPOMI X_{CO} retrievals for the mean posterior flux across the inversion ensemble for each inventory is shown as maps in Fig. S8 and as histograms in Fig. S9. The prior inventories indicate large-scale positive biases, suggesting that the model-simulated X_{CO} levels are underestimated. This implies that prior emissions are too low to match observed atmospheric X_{CO} abundances. The prior estimates show both regionally positive biases (QFED) and negative biases (GFED) over Canada during May–Sep 2023. The posterior fluxes largely mitigate these biases, but leave small negative biases over North America and small positive biases elsewhere. A similar spatial structure is seen when the emissions are released at a modeled injection height (IS4FIRES), suggesting this does not explains the residual structures. Comparisons of the mean posterior flux across the inversion ensemble for each inventory against the East Trout Lake (Fig. S10) and Park Falls (Fig. S11) TCCON sites show marked improvements for the posterior X_{CO} relative to the prior X_{CO}. Considerable scatter remains, particularly at

elevated X_{CO} mole fractions above 200 ppb. In part, this results from the inability of the transport model to accurately model and represent biomass burning plumes.

Tables 1–3 show the mean and standard deviation against TROPOMI, East Trout Lake, and Park Falls for each individual inversions. These statistics are shown for forward simulations where the emissions are released at the surface (as in the inversion) or at a fire injection height that is modeled by IS4FIRES. We find that the posterior observation-model differences to be much smaller that the standard deviation of the mismatch for all cases. The relative performance of individual inversion configurations are not clearly distinguishable, as they all show similar agreement against TCCON data.

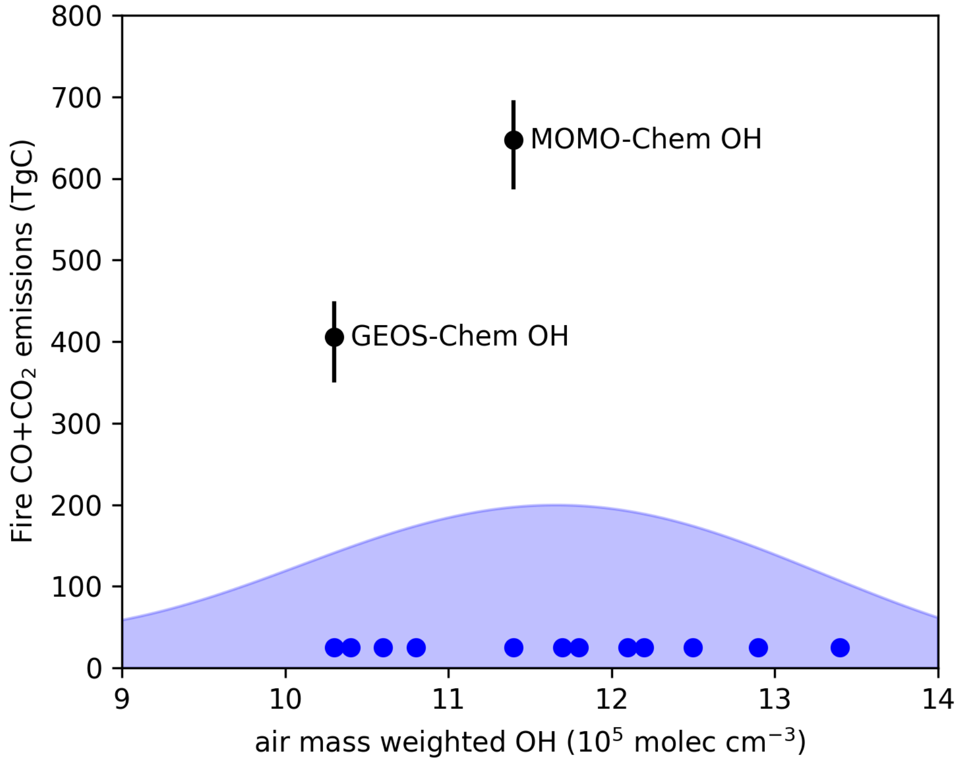

Fig. S1 Sensitivity of $CO+CO_2$ emission estimate to prescribed OH abundances. Top-down May-Sep $CO+CO_2$ emissions against the air-mass-weighted OH abundance for the GEOS-Chem and MOMO-Chem OH fields, with error bars showing the range of estimates due to inversion configuration. The distribution of modeled OH abundances for the Atmospheric Chemistry and Climate Modeling Intercomparison Project (ACCMIP) are shown by the blue dots, and a gaussian density function of these dots is shown by the shaded blue area.

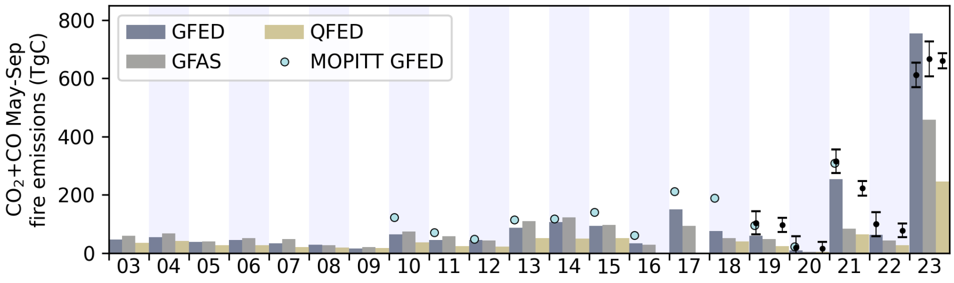

Fig. S2 May-Sep forest fire CO_2+CO emissions over 2003–2023. Bottom-up estimates are shown by the bars for GFED, GFAS, and QFED. Top-down estimates that assimilate MOPITT X_{CO} are shown in green. Estimates that assimilate TROPOMI X_{CO} are shown with error bars. There was an error in the GFAS inversions during 2019-22, so those are not shown.

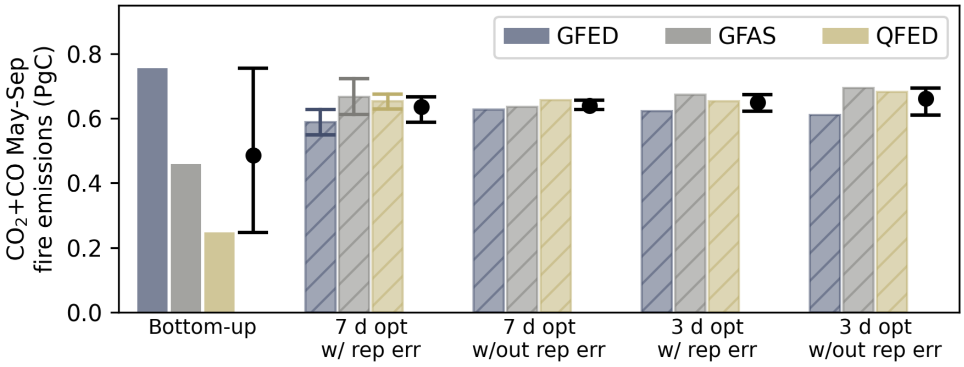

Fig. S3 Maximum a posteriori CO+CO_2 fire emission over Canadian forests for May-Sep 2023 using four different inversion configurations. From left-to-right, posterior estimates are shown for inversions configuration (i)–(iv). The posterior uncertainties (1 σ) for experiment a are also shown. The black error bars show the range in maximum a posteriori estimates but do not include the Bayesian errors.

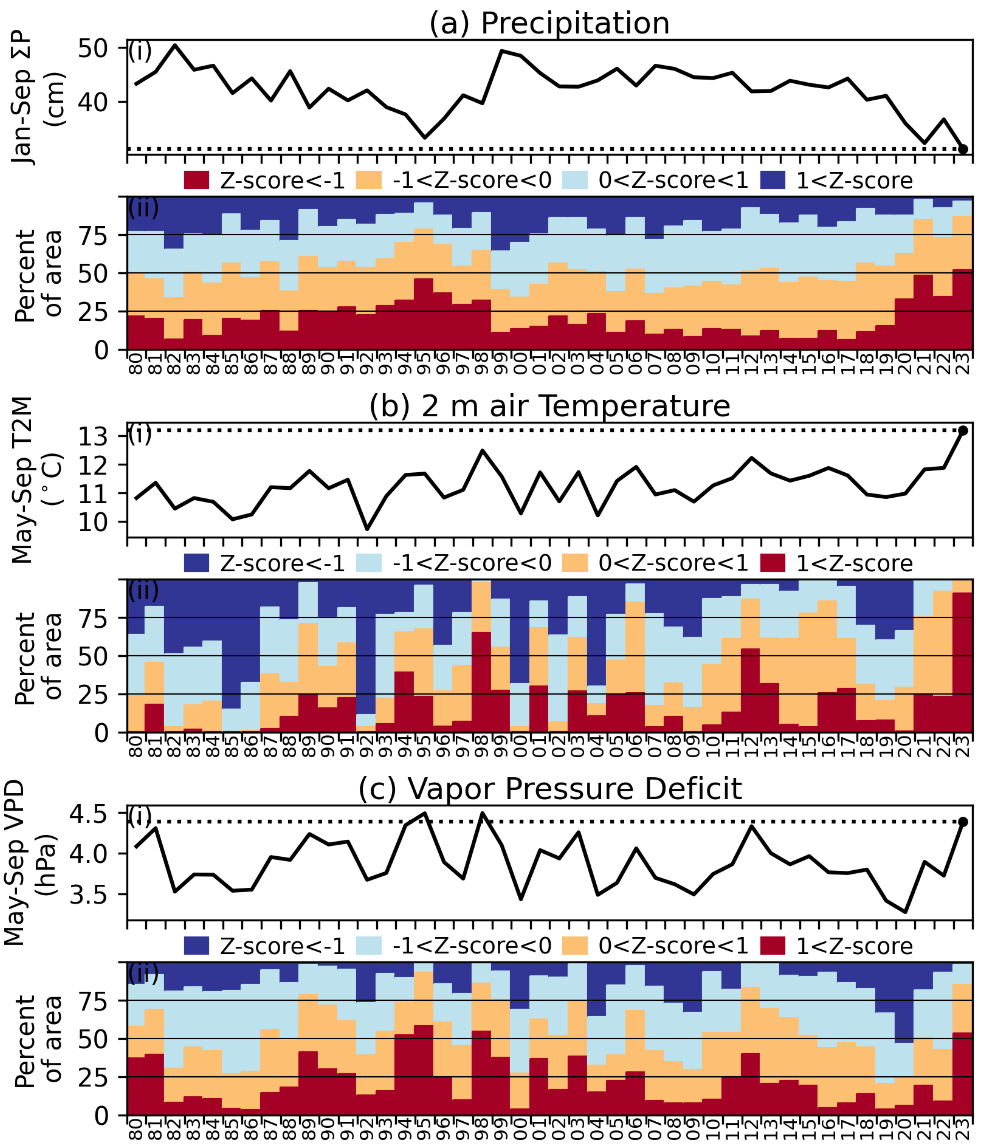

Fig. S4 Climate variations for Canadian forests over 1980–2023 for (a) precipitation, (b) 2 m air temperature, and (c) VPD. Panel (i) shows the timeseries of mean values over Canadian forests while panel (ii) shows the percent of area with each Z-score

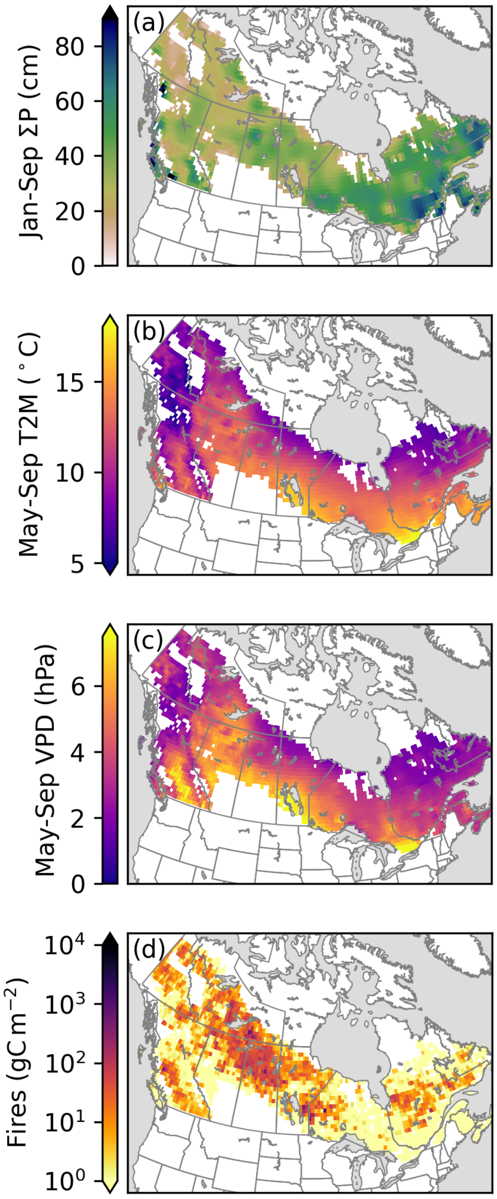

Fig. S5 2003-2022 Climatology for Canadian forests. Maps of (a) CPC Global Unified Gauge-Based cumulative precipitation, (b) MERRA-2 2 m temperature, (c) MERRA-2 VPD, and (d) fire CO_2+CO emissions from the GFED4.1s database. All maps are shown at a spatial resolution of 0.5° × 0.625°

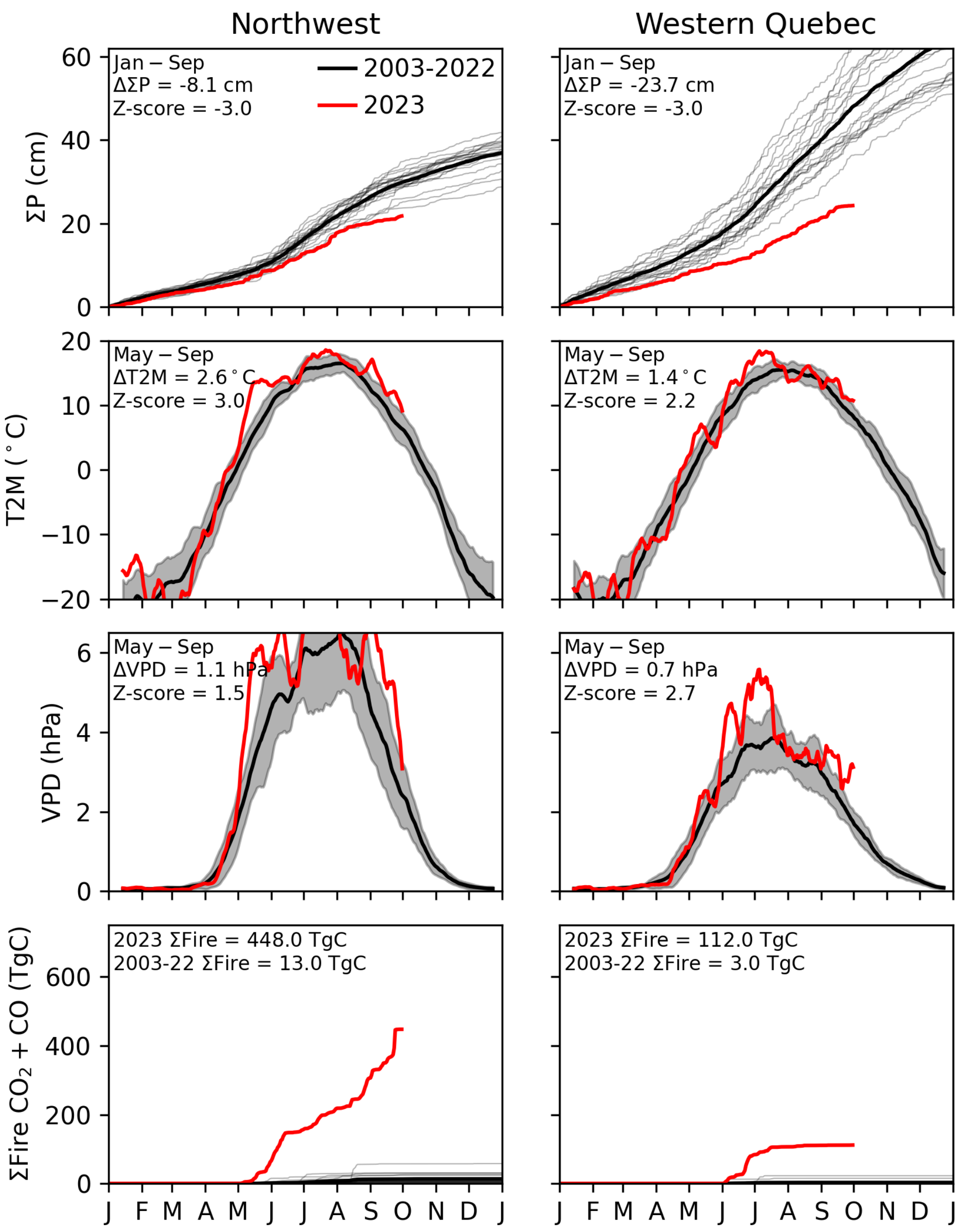

Fig. S6 Forest climate anomalies during 2023 over two regions: Northwestern Canada near Great Slave Lake (57–62N,110-125W), and western Quebec (49–55 N, 72–80 W). Timeseries of (top-to-bottom) CPC Global Unified Gauge-Based cumulative precipitation, MERRA-2 2 m temperature (with 2-week running mean), MERRA-2 VPD (with 2-week running mean), and fire CO_2+CO emissions from the GFED4.1s database.

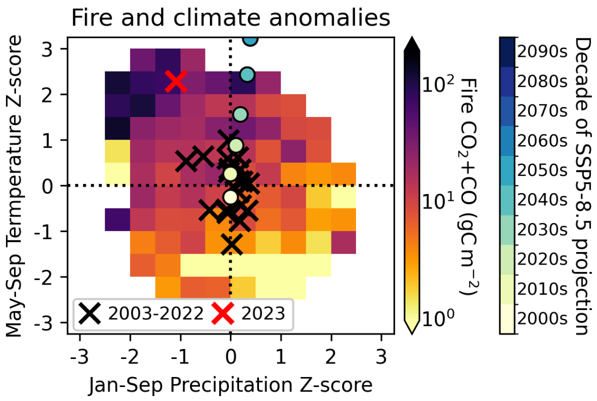

Fig. S7 Same as Fig. **??** but for CMIP6 SSP5-8.5.

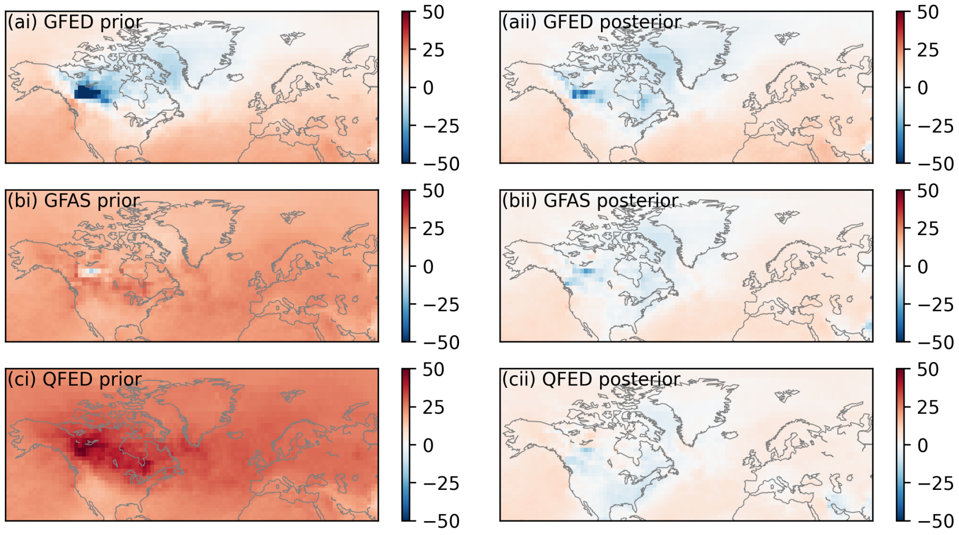

Fig. S8 Map of May-Sep 2023 mean TROPOMI X_{CO} retrievals minus model simulated X_{CO}. The difference is shown for the (ai) GFED4.1s inventory, (aii) mean posterior with GFED4.1s as a prior, (bi) GFAS inventory, (bii) mean posterior with GFAS as a prior, (ci) QFED inventory, and (cii) mean posterior with QFED as a prior.

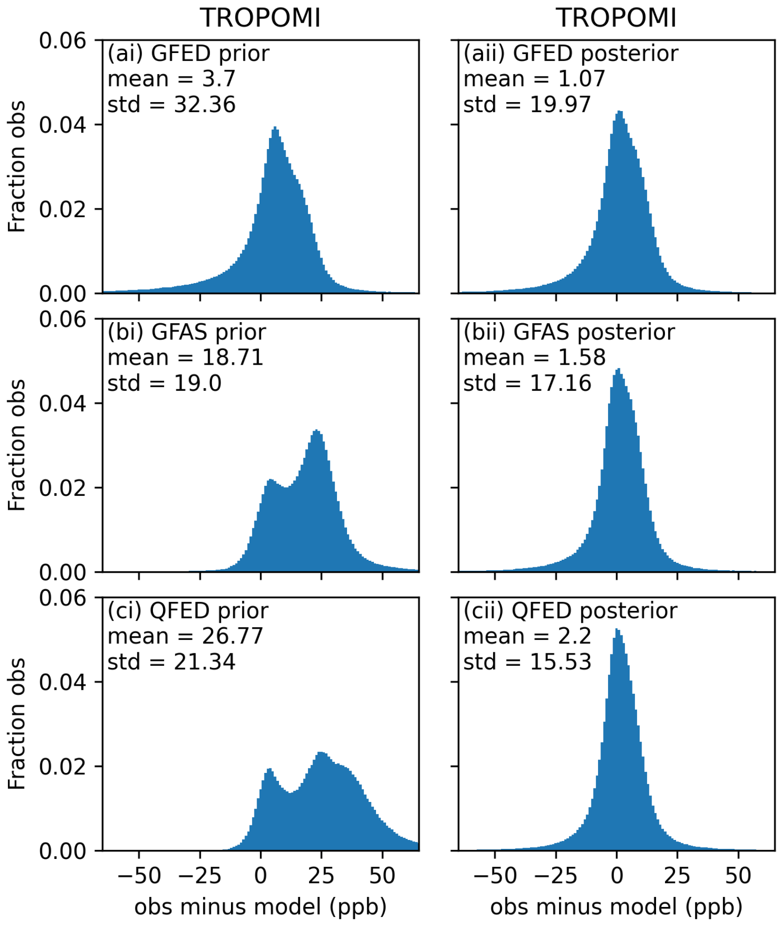

Fig. S9 Histograms of TROPOMI X_{CO} retrievals minus model simulated X_{CO} over the region shown in Fig. S8 (20–86 °N, 170 °W–75 °E). Histograms are shown for the (ai) GFED4.1s inventory, (aii) mean posterior with GFED4.1s as a prior, (bi) GFAS inventory, (bii) mean posterior with GFAS as a prior, (ci) QFED inventory, and (cii) mean posterior with QFED as a prior. The mean and standard deviations are shown in the top-left corner for each panel.

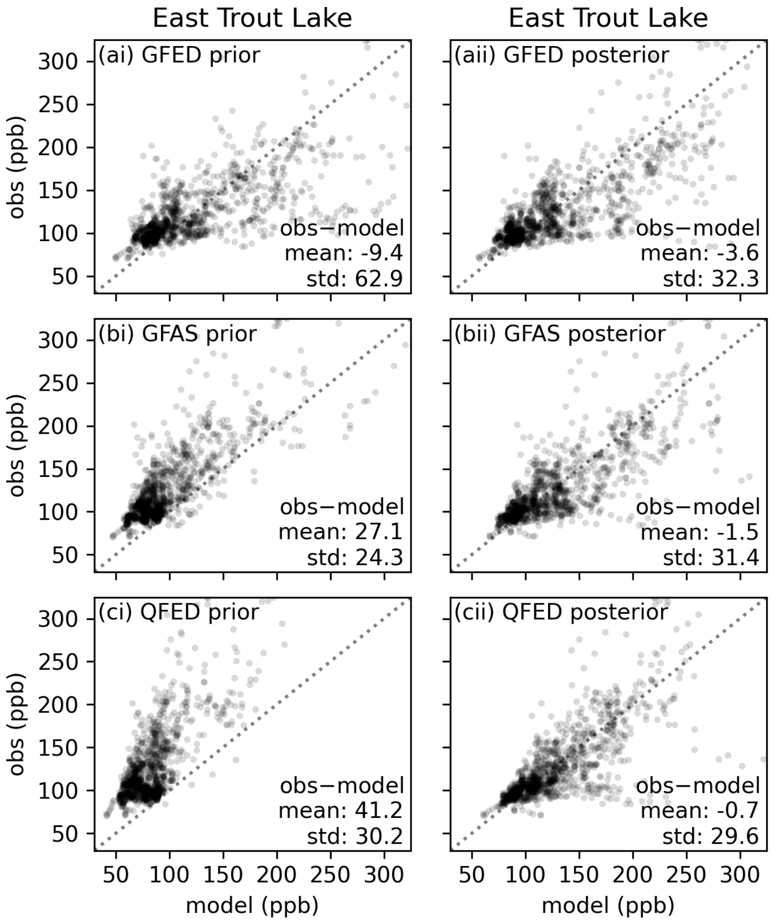

Fig. S10 TCCON X_{CO} retrievals against model simulated X_{CO} at East Trout Lake (54.35 °N, 104.99 °W). The comparison is shown for the (ai) GFED4.1s inventory, (aii) mean posterior with GFED4.1s as a prior, (bi) GFAS inventory, (bii) mean posterior with GFAS as a prior, (ci) QFED inventory, and (cii) mean posterior with QFED as a prior. Statistics on the TCCON minus model differences are given in the lower right of each panel.

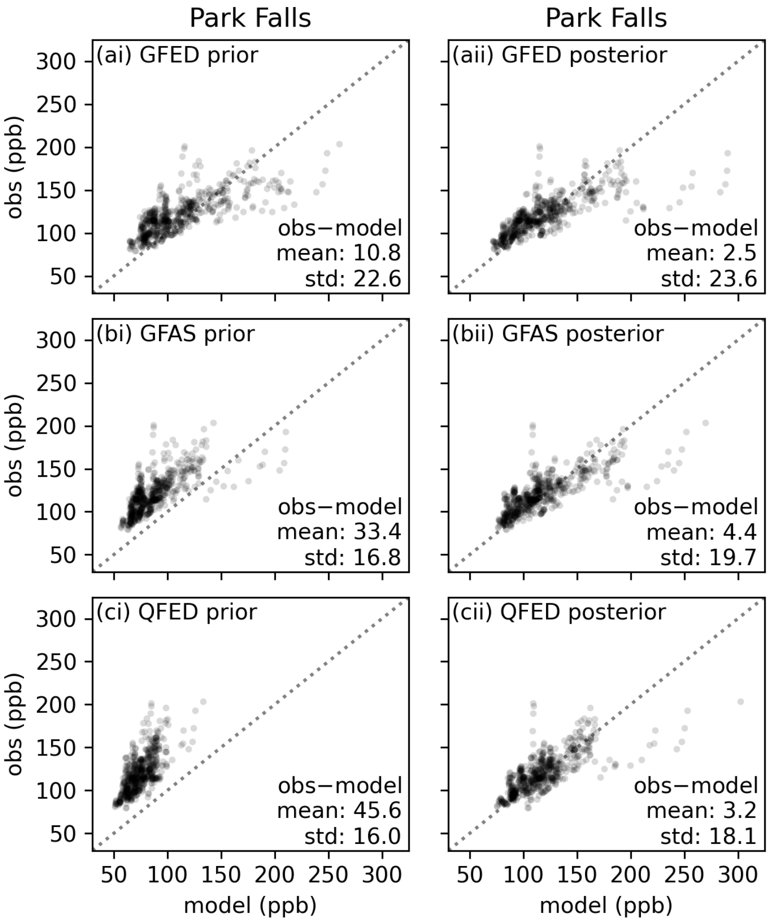

Fig. S11 TCCON X_{CO} retrievals against model simulated X_{CO} at Park Falls (45.95 °N, 90.27 °W). The comparison is shown for the (ai) GFED4.1s inventory, (aii) mean posterior with GFED4.1s as a prior, (bi) GFAS inventory, (bii) mean posterior with GFAS as a prior, (ci) QFED inventory, and (cii) mean posterior with QFED as a prior. Statistics on the TCCON minus model differences are given in the lower right of each panel.

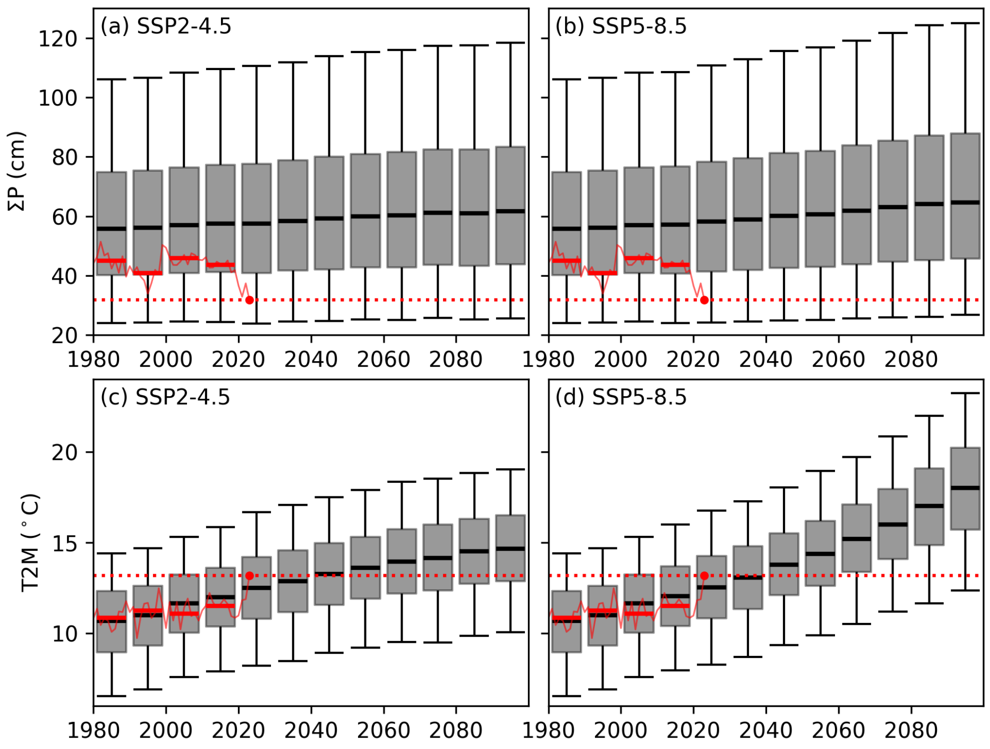

Fig. S12 Box-and-whisker plots of the decadal mean CMIP6 cumulative precipitation (Jan-Sep) and mean T2M (May-Sep) over Canadian boreal forests. The black line shows the ensemble median, shaded grey area shows the interquartile range, and error bars show the 5–95 percentile range. The decadal means and individual years of the CPC Global Unified Gauge-Based Analysis of Daily Precipitation data and MERRA-2 T2M data are shown in red. The MERRA-2 T2M is shown to correspond closely with the CMPI6 ensemble median while the Precipitation data is around the 25th percentile of the CMIP6 ensemble, suggesting most CMIP6 models overestimate precipitation over the Canadian boreal forests.

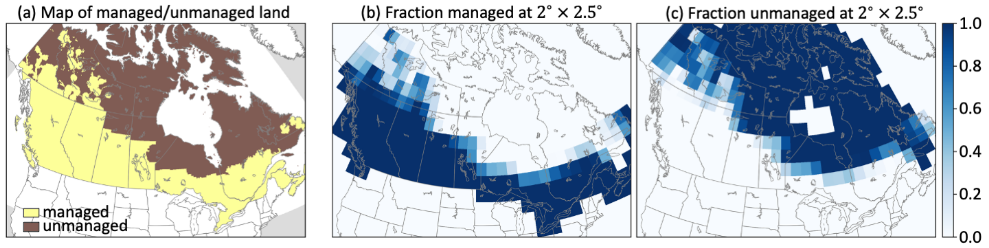

Fig. S13 (a) Managed and unmanaged lands within Canada. Fraction of inversion model $2° \times 2.5°$ grid cells that are (a) managed or (b) unmanaged lands.

Table 1 Mean ± standard deviation (ppb) of the observation minus model simulated TROPOMI X_{CO} over the region 20–86 °N and 170 °W–75 °E during May-Sep 2023. Statistics are shown for the prior inventories and maximum a posteriori flux estimates for the four inversion configurations, and are shown for both release at model surface and IS4FIRES plume injection height.

	surface release			injection release		
Experiment	GFED	GFAS	QFED	GFED	GFAS	QFED
prior	3.7 ± 32.4	18.7 ± 19.0	26.8 ± 21.3	3.4 ± 32.0	13.7 ± 18.3	25.4 ± 20.6
3-day/no-rep	3.2 ± 19.1	2.4 ± 18.1	2.4 ± 15.7	2.8 ± 19.9	3.2 ± 18.0	2.3 ± 15.9
3-day/rep	0.1 ± 23.2	0.6 ± 18.0	1.9 ± 16.1	0.2 ± 22.1	1.1 ± 17.9	1.9 ± 16.1
7-day/no-rep	0.9 ± 20.2	2.5 ± 16.7	2.3 ± 15.6	-0.2 ± 20.5	5.0 ± 16.4	2.2 ± 15.6
7-day/rep	0.1 ± 23.2	0.6 ± 18.0	1.9 ± 16.0	-0.2 ± 21.4	1.2 ± 17.7	1.9 ± 15.7
mean	1.1 ± 20.0	1.6 ± 17.1	2.2 ± 15.5	0.6 ± 19.6	2.6 ± 16.8	2.1 ± 15.5

Table 2 Mean ± standard deviation (ppb) of the observation minus model simulated TCCON X_{CO} at East Trout Lake. Statistics are shown for the prior inventories and maximum a posteriori flux estimates for the four inversion configurations, and are shown for both release at model surface and IS4FIRES plume injection height.

	surface release			injection release		
Experiment	GFED	GFAS	QFED	GFED	GFAS	QFED
prior	-9.4 ± 62.9	27.1 ± 24.3	41.2 ± 30.2	-8.6 ± 58.7	23.0 ± 24.9	40.0 ± 31.2
3-day/no-rep	0.5 ± 29.5	-0.2 ± 31.6	-2.5 ± 31.8	-0.5 ± 34.2	0.1 ± 32.8	-3.0 ± 34.2
3-day/rep	-3.2 ± 34.0	-3.4 ± 34.5	1.8 ± 26.4	-4.5 ± 37.8	-3.7 ± 35.9	1.1 ± 30.1
7-day/no-rep	-7.4 ± 37.2	-1.0 ± 32.3	-1.8 ± 32.4	-10.2 ± 45.2	1.3 ± 35.8	-2.5 ± 33.8
7-day/rep	-4.2 ± 35.6	-1.5 ± 31.6	-0.4 ± 30.5	-3.3 ± 37.5	-1.9 ± 33.8	-1.0 ± 31.3
mean	-3.6 ± 32.3	-1.5 ± 31.4	-0.7 ± 29.6	-4.6 ± 36.1	-1.1 ± 33.5	-1.3 ± 31.6

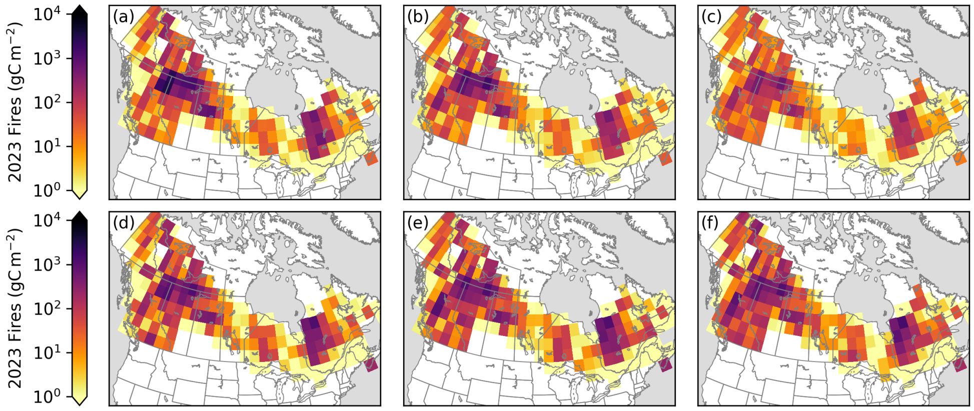

Fig. S14 Spatial distribution of May-Sep 2023 (a-c) prior and (d-e) posterior $CO+CO_2$ fire emissions at 2° × 2.5° spatial resolution.

Table 3 Mean ± standard deviation (ppb) of the observation minus model simulated TCCON X_{CO} at Park Falls. Statistics are shown for the prior inventories and maximum a posteriori flux estimates for the four inversion configurations, and are shown for both release at model surface and IS4FIRES plume injection height.

	surface release			injection release		
Experiment	GFED	GFAS	QFED	GFED	GFAS	QFED
prior	10.8 ± 22.6	33.4 ± 16.8	45.6 ± 16.0	9.8 ± 23.3	25.3 ± 16.8	42.9 ± 15.8
3-day/no-rep	5.3 ± 26.6	6.2 ± 19.7	3.1 ± 17.9	5.2 ± 28.5	6.4 ± 19.9	2.6 ± 18.0
3-day/rep	2.1 ± 27.5	1.8 ± 21.8	3.9 ± 19.9	1.8 ± 27.3	2.8 ± 19.3	3.7 ± 20.0
7-day/no-rep	0.0 ± 21.1	5.9 ± 17.9	3.0 ± 17.5	0.0 ± 19.8	9.8 ± 16.7	2.6 ± 16.8
7-day/rep	2.5 ± 24.3	3.7 ± 22.5	2.9 ± 18.6	2.0 ± 26.9	4.0 ± 20.9	2.5 ± 18.4
mean	2.5 ± 23.6	4.4 ± 19.7	3.2 ± 18.1	2.3 ± 24.3	5.7 ± 18.6	2.9 ± 18.0

References

[1] Miyazaki, K., Bowman, K.W., Yumimoto, K., Walker, T., Sudo, K.: Evaluation of a multi-model, multi-constituent assimilation framework for tropospheric chemical reanalysis. Atmospheric Chemistry and Physics **20**(2), 931–967 (2020) https://doi.org/10.5194/acp-20-931-2020

[2] Miyazaki, K., Bowman, K., Sekiya, T., Eskes, H., Boersma, F., Worden, H., Livesey, N., Payne, V.H., Sudo, K., Kanaya, Y., Takigawa, M., Ogochi, K.: Updated tropospheric chemistry reanalysis and emission estimates, tcr-2, for 2005–2018. Earth System Science Data **12**(3), 2223–2259 (2020) https://doi.org/10.5194/essd-12-2223-2020

[3] Park, R.J., Jacob, D.J., Field, B.D., Yantosca, R.M., Chin, M.: Natural and transboundary pollution influences on sulfate-nitrate-ammonium aerosols in the united states: Implications for policy. Journal of Geophysical Research: Atmospheres **109**(D15) (2004)

[4] Voulgarakis, A., Naik, V., Lamarque, J.-F., Shindell, D.T., Young, P.J., Prather, M.J., Wild, O., Field, R.D., Bergmann, D., Cameron-Smith, P., Cionni, I., Collins, W.J., Dalsøren, S.B., Doherty, R.M., Eyring, V., Faluvegi, G., Folberth, G.A., Horowitz, L.W., Josse, B., MacKenzie, I.A., Nagashima, T., Plummer, D.A., Righi, M., Rumbold, S.T., Stevenson, D.S., Strode, S.A., Sudo, K., Szopa, S., Zeng, G.: Analysis of present day and future oh and methane lifetime in the accmip simulations. Atmospheric Chemistry and Physics **13**(5), 2563–2587 (2013) https://doi.org/10.5194/acp-13-2563-2013
